# An Adaptive Parameterized Domain Mapping Method and Its Application in Wheel–Rail Coupled Fault Diagnosis for Rail Vehicles

**DOI:** 10.3390/s23125486

**Published:** 2023-06-10

**Authors:** Zihang Xu, Jianwei Yang, Dechen Yao, Jinhai Wang, Minghui Wei

**Affiliations:** 1 Beijing Key Laboratory of Performance Guarantee on Urban Rail Transit Vehicles, Beijing University of Civil Engineering and Architecture, Beijing 100044, China; xzh979797@126.com (Z.X.); yaodechen@bucea.edu.cn (D.Y.); wangjinhai@bucea.edu.cn (J.W.); 2108550020059@stu.bucea.edu.cn (M.W.); 2 School of Mechanical-Electronic and Vehicle Engineering, Beijing University of Civil Engineering and Architecture, Beijing 100044, China

**Keywords:** rail vehicles, parameterized domain mapping, wheel–rail coupled fault, fault diagnosis, whale optimization algorithm

## Abstract

The rapid development of cities in recent years has increased the operational pressure of rail vehicles, and due to the characteristics of rail vehicles, including harsh operating environment, frequent starting and braking, resulting in rails and wheels being prone to rail corrugation, polygons, flat scars and other faults. These faults are coupled in actual operation, leading to the deterioration of the wheel–rail contact relationship and causing harm to driving safety. Hence, the accurate detection of wheel–rail coupled faults will improve the safety of rail vehicles’ operation. The dynamic modeling of rail vehicles is carried out to establish the character models of wheel–rail faults including rail corrugation, polygonization and flat scars to explore the coupling relationship and characteristics under variable speed conditions and to obtain the vertical acceleration of the axle box. An APDM time–frequency analysis method is proposed in this paper based on the PDMF adopting Rényi entropy as the evaluation index and employing a WOA to optimize the parameter set. The number of iterations of the WOA adopted in this paper is decreased by 26% and 23%, respectively, compared with PSO and SSA, which means that the WOA performs at faster convergence speed and with a more accurate Rényi entropy value. Additionally, TFR obtained using APDM realizes the localization and extraction of the coupled fault characteristics under rail vehicles’ variable speed working conditions with higher energy concentration and stronger noise resistance corresponding to prominent ability of fault diagnosis. Finally, the effectiveness of the proposed method is verified using simulation and experimental results that prove the engineering application value of the proposed method.

## 1. Introduction

### 1.1. Motivation and Incitement

In recent years, China’s urban rail transit has developed rapidly, and rail vehicles have become the mainstay of intercity transportation. The vehicle’s operating speed, routes and mileage have been constantly increasing to satisfy the demand for more trips. However, rail vehicles’ operation has more special characteristics: frequent load changes, short start–stop cycles, multiple interference sources, frequent speed fluctuations and shock load, etc. These characteristics easily lead to rail corrugation, abrasion, cracking, and stripping of rail, with concurrent polygonization, flat scars, spalling abrasions and other faults in wheels. These faults affect the safety of vehicle operation, seriously reducing the operating efficiency of rail vehicles and passenger comfort [1,2,3,4,5,6,7].

Although domestic research on fault diagnosis of urban rail vehicles has been conducted in great depth, most current research is aimed at the diagnosis of a single fault such as rail corrugation, wheel polygons, flat scars, etc. Yet there is limited research on the signal processing of coupled faults such as rail corrugation–polygon fault. Coupled faults cause greater deterioration in the vibration stability of the vehicle body compared with a single fault. The existence of rail corrugation degenerates the dynamic performance of rail vehicles under a single fault, such as polygons and flat scars, which leads to increased complexity in the axle box’s acceleration signal characteristics. Additionally, vibration signals generated under the action of coupled faults are usually nonstationary signals with severely changing IFs. Therefore, there is an urgent need for a diagnostic method with excellent performance and strong noise resistance for handling nonstationary signals generated by rail vehicles under coupled faults.

### 1.2. Literature Review

Certain progress has been made on research of wheel–rail faults so far. Xu et al. [8] proposed a method for dynamic quantitative diagnosis and estimation of rail corrugation according to acceleration data of high-speed comprehensive inspection trains. They applied the inverse synchrosqueezing STFT method to estimate the amplitude of rail corrugation, achieving a dynamic quantitative diagnosis of rail corrugation. SUDAY et al. [9] proposed a novel method for detecting rail corrugation using wavelet analysis of axle box acceleration, which can detect the exact location and frequency range of rail corrugation. Chen [10] constructed a rigid–flexible coupled dynamic model considering flexible wheel pairs and derived the time domain and frequency domain characteristics of the axle box’s acceleration under wheel polygon excitation by analyzing the polygon spectra of different orders in the frequency domain, based on which a wheel polygon identification process was proposed, which can effectively detect the order of a wheel polygon. Yang [11] extracted the energy values of the vertical vibration acceleration of different components as the judging index. Then, they compared the characteristics of acceleration signal both in the time domain and in the energy of the components including the car body, frame, axle box, wheel pair and rail for flat scar detection. However, most of the faults in rail vehicles during operation are coupled instead of appearing separately, resulting in the generation of wheel–rail coupled faults. Among them, Xu et al. [12] learned the interaction between wheel polygons and rail corrugation of high-speed railroads through establishing a wheel–rail contact model which verified that the influence of wheel polygons on the friction-coupled vibration of the wheel–rail system is greater than that of rail corrugations. Liu et al. [13] established a multibody dynamic model upon which they conducted a high-speed operation test and the corresponding dynamic simulation of the wheelset. They determined that the influences on vibration amplitude of the axle box generated by wheel polygonization, wheel–rail polygonal wear and rail corrugation are 26.7%, 48.3% and 25%, respectively. Therefore, it can be concluded that vehicle dynamic response signals including axle box acceleration play an important role in the detection of rail corrugation, wheel polygons and flat scars. It is of great urgency and necessity to learn about complex wheel–rail coupled faults.

The existing time–frequency analysis methods include STFT, WT, Wigner–Ville distribution, etc. STFT selects a time–frequency-localized pseudo-smooth window function so that the signal to be processed is smooth in that period to obtain the signal power spectrum. However, it is difficult for STFT to reconcile the needs between high time resolution for severely varied signals and high-frequency resolution for components with lower frequency. To address this problem, researchers have proposed several improved STFT-based algorithms with adaptive properties enabling them to handle nonstationary signals [14,15,16,17,18]. Wei et al. [19] proposed a detection method for bearing faults that combines empirical mode decomposition and adaptive time-varying parameter short time Fourier synchronous compression transform to solve the adaptive problem of signals under various operating conditions. STFT based on the inner product principle is capable of detecting the harmonic features of rotating machinery faults. However, it difficult to improve the energy concentration of TFR for nonstationary signals with fast time-varying frequency components [20]. To overcome this drawback, Huang et al. [21] proposed a sparse TFA method combining sparse time–frequency analysis with IF estimation based on the basic weighted sparse model for analyzing nonstationary signals with large IF variations, which improves both energy concentration and accuracy. Hu et al. [22] proposed an adaptive time domain signal segmentation method that adopts cubic spline interpolation to envelope the original signal. This method can effectively suppress the high amplitude components of vibration signals generated by the subway gearbox and extract weak fault features caused by uniform wear of the gearbox in both the time and frequency domains. Shi et al. [23] proposed a simultaneously compressed fractional order wavelet transform to handle signals with rapidly changing IFs.

The methods discussed above are nonparameterized time–frequency analysis methods that are mainly applied to process signals with special patterns [24] yet not suitable for handling nonstationary signals. Therefore, nonstationary to stationary mapping techniques have been under extensive research in recent years. GD is a powerful tool proposed by Olhede [25] to handle nonstationary signals with frequently changing IFs. Huang et al. [26] proposed a method combined with time–frequency squeezing and GD without resampling to realize the diagnosis of variable speed bearing faults. Ma et al. [27] proposed a diagnosis method for rolling bearing faults on the basis of adaptive GD that greatly improves the fault identification rate. Liu et al. [28] proposed a novel flexible GD-based method mapping time-varying frequencies of extensive samples under variable speed conditions to the defined base frequency as well as its multiples.

Additionally, TLOT is another significant tool proposed by Bonnardot [29] for mapping nonstationary signals to stationary ones, which overcomes the tachometer-necessary limitation of the traditional order tracking technique. Wu et al. [30] proposed an improved TLOT method based on nonlinear compensation demodulation transform for the appliance of fault diagnosis of mechanical extreme state monitoring. A TLOT-based method combined with time varying filtering and continuous WT was proposed by Jing [31], which could be applied under the condition of strong fluctuations in speed with strong noises. Wan et al. [32] proposed a variational modal decomposition filtering- and synchronous extraction transformation-based TLOT method that achieved an improvement in IF estimation accuracy.

In addition, parameterized TFA is another essential tool for handling nonstationary signals. Yang et al. [33,34] first proposed a GPTF for parameterized time–frequency analysis that has the significant advantage of customizing the generalized kernel to characterize the time–frequency characteristics of nonstationary signals accurately. Various parameterized time–frequency transforms can be implemented from the same perspective by replacing the kernel function that provides the availability of a single generalized time–frequency transform for applications with signals of different features. On this basis, a parameterized time–frequency analysis method for analyzing nonstationary vibration signals generated by variable-speed rotating machinery was proposed. This method showed superior performance in feature extraction accuracy compared to other traditional TFA methods and has good application prospects for parameter identification and fault diagnosis. Deng et al. [35] proposed a parameter identification method for nonlinear systems based on the polynomial Chirplet transform, which is a powerful tool for handling nonstationary signals. Chen et al. [36] proposed an algorithm called Chirplet path fusion for analyzing nonstationary signals with time-varying frequencies that had better IF extraction capability in a noisy environment and could be applied to situations with short time signals. Wang et al. [37] proposed a diagnosis method for rotor bumper faults based on nonlinear compressive time–frequency transformation that could better extract the rapidly oscillating period IFs in the vibration signal generated by rotor bumper faults. Zhou et al. [38] proposed an effective nonstationary signal analysis method based on GPTF and a multicomponent instantaneous frequency extraction method that was superior to traditional time–frequency analysis methods and could be applied to feature extraction of large rotating machinery for condition monitoring and fault diagnosis. Li et al. [39] proposed a parameterized resampling time–frequency transformation method that effectively improved the time–frequency resolution of nonstationary multicomponent signals. Li et al. [40] proposed the PDM method, which constructs a pseudo-time domain by setting the PDMF, thus realizing the order tracking of vibration signals generated by rotating mechanical parts, which solved the frequency distortion problem and achieved excellent anti-noise performance. The parameterized TFR adopts additional parameters closely related to the signal under consideration and is able to produce a TFR with higher energy concentration compared to the nonparameterized TFR [41].

The parameterized TFA method is capable of obtaining a better effect through parameter setting. However, signals in actual working condition are complex, and it is difficult to set the parameters effectively, which limits parameterized TFA’s application in practical engineering. Researchers have carried out much work on optimization. Li et al. [40] found the optimal parameter set through adopting PSO by maximizing the fitness. Chen et al. [42] proposed a discrete gray wolf optimization algorithm that could be used for solving binary problems and broadening its practical application areas. Bai et al. [43] proposed a new spectral Markov transfer field algorithm by constructing a first-order Markov transfer matrix of frequency domain signals that represented the spectral features of vibration signals in the form of images and solved the problem that traditional algorithms were not applicable in the frequency domain. He et al. [44] proposed an integrated transmission neural network for the problem of automatic diagnosis of fault types in rotating machinery under different operating conditions that fully combined the characteristics of deep learning, migration learning and integrated learning. Shao et al. [45] proposed a variable-speed rotor-bearing system fault diagnosis method based on two-level parameter transfer and infrared thermal images that improved the diagnostic capability and adaptability of the fault diagnosis method for rotor-bearing systems at variable speeds. Bai et al. [46] proposed a new frequency domain Gramian angular field algorithm that encoded wheel plane vibration signals into feature images, achieving accurate diagnostic results with high separability. Wang et al. [47] proposed an inheritance decision making method that combined dislocation time–frequency representation with pretrained convolutional neural networks. Additionally, they compared continuous WT and synchrosqueezed transform to STFT methods for intelligent diagnosis. Chu et al. [48] proposed a fault diagnosis method for rolling bearings based on the SSA variational modal decomposition parameters combined with K-mean singular value decomposition that adopted SSA upon iterative optimization search to accurately extract rolling bearing faults in a low signal-to-noise ratio environment. The problem addressed in this paper is the establishing a method for optimizing the parameter set of the PDMF with improved self-adaptability.

### 1.3. Contribution and Paper Organization

The main contributions and novelty of the paper are listed as follows:

(1) The purpose of this paper is to diagnose wheel–rail coupled faults. Vibration signals of an axle box under coupled faults are more complex than those under single faults. Hence, it is difficult to distinguish separate faults from vibration signals of an axle box under coupled faults using traditional methods.
(2) Rényi entropy is taken as an optimization objective to evaluate the performance of the method proposed in this paper to improve the energy concentration of TFR.
(3) A comparison is carried out between PSO, SSA and WOA to evaluate their performance during the optimization process. The WOA adopted in this paper achieves higher accuracy at a faster speed.
(4) Compared with STFT and PDM, the APDM proposed in this paper has a better diagnostic effect and better performance, including higher energy concentration and stronger noise resistance.

The rest of this paper is structured as follows: Section 2 establishes a dynamic model of rail vehicles based upon which fault models including rail corrugation, polygonization and flat scarring are established, respectively. Section 3 proposes the APDM method and introduces the method’s implementation steps and principles in detail. Section 4 analyzes the characteristics of coupled faults by simulating different fault types including rail corrugation, polygonization and flat scar under variable speed conditions. Then, the APDM proposed in Section 3 is adopted to perform the time–frequency analysis to achieve the accurate localization of fault characteristics and frequencies. Section 5 concludes that APDM realizes excellent diagnostic effect for different wavelengths of rail corrugation, orders of polygon, and flat scars under variable speed working conditions, which is verified using simulated and field-tested data. Accurate positioning of coupled fault frequencies is achieved using APDM with outstanding performance including strong noise resistance, high time–frequency resolution and high energy concentration, which provides a reference for the diagnosis of wheel–rail coupled faults in rail vehicles under multiple working conditions and has certain engineering application value.

## 2. Models of Rail Vehicles and Wheel–Rail Faults

A dynamic model of rail vehicles is established in this section upon which the fault models including rail corrugation, polygonization and flat scar are established. Additionally, the excitation frequency of each fault is derived.

### 2.1. Dynamic Model of Rail Vehicles

**Figure 1:**
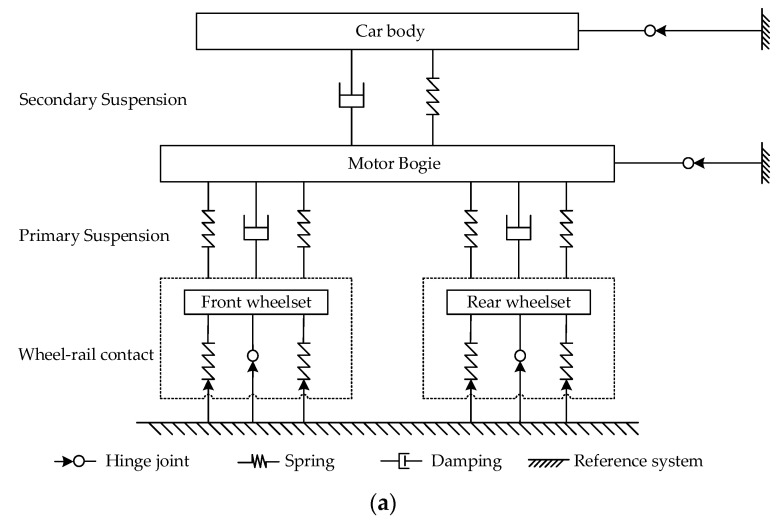

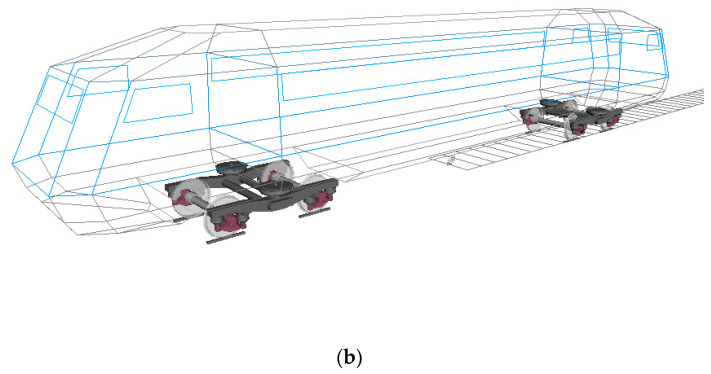
Dynamic model of rail vehicles: (**a**) topology of vehicle multibody system; (**b**) dynamic model of vehicle system.

Careful consideration should be paid to the system components’ physical characteristics and analysis of their spatial motion mechanical behavior during the modeling process of rail vehicles. A reasonable connection relationship between the components of the force element system is established by defining proper articulation constraints. To visualize the number of components in the vehicle system and the reference interface of interacting force elements, a topology diagram must be constructed before modeling to clarify how the system components are interrelated. Figure 1a,b show the topology and the dynamic model of rail vehicles, respectively.

### 2.2. Fault Modeling

#### 2.2.1. Rail Corrugation

Rail corrugation is a common harmonic type of excitation disturbance in rails, calculation using Equation (1).

(1) $$Z_{0}\left(t\right)=\frac{1}{2}a\left(1-\cos\omega \cdot t\right),\left(t\leq \frac{\lambda }{v}\right)$$

(2) $$\omega =\frac{2\pi v}{\lambda }$$

where *λ* and *a* are the wavelength and wave depth of the rail corrugation, respectively, and *v* is the vehicle speed. The excitation frequency function of rail corrugation is

(3) $$f=\frac{v}{\lambda }$$

The modeling of wheel polygonization is established in the next section.

#### 2.2.2. Wheel Polygon

The simple harmonic function demonstrated in Equation (4) represents the altering trend of a polygonal wheel.

(4) $$\Delta R\left(\beta \right)=A\sin\left[n\left(\beta +\beta_{0}\right)\right]$$

where ∆*R* is the wheel diameter difference, *β* is the wheel rotation angle, *β*_0_ is the initial phase offset, *A* and *n* are wave depth and order of the wheel polygon, respectively.

According to actual measurements of wheel polygons on site, the main order components of rail vehicles’ wheels are concentrated below 10th order and the wave depth can reach about 0.1 mm. Among them, the 1st to 4th orders are the initial polygonal phenomena caused by the manufacturing and maintenance process of wheels. With the accumulation of vehicle running time, polygons of other orders gradually appear, causing significant enhancement in rail–wheel cycling impulse effect. Wheel polygons of the 8th and 9th orders are under research in this paper and the wave depth is set at 0.12 mm.

The excitation frequency function of a polygon fault is shown in Equation (5).

(5) $$f=\frac{nv}{2\pi R}$$

where *n* is the polygon order, *v* is the vehicle speed and *R* is the wheel radius.

#### 2.2.3. Flat Scar

Flat scar faults can be divided into two types: the ideal new flat scar, and the old flat scar after the edges and corners of the scratch are rounded. However, new flat scar faults quickly transform into old flat scars after wear and tear. According to field testing, it was found that the inspection results of the repaired rail vehicles were all old flat scars, and no ideal new flat scars were found. Therefore, this paper focuses on the excitation response characteristics of old flat scars. The excitation of old flat scars can be described using Equation (6).

(6) $$Z_{P}=\frac{1}{2}h\left[1-\cos\left(2\pi x/L\right)\right]$$

(7) $$h=\frac{L^{2}}{16R}$$

where *L* is the length of the flat scar, *R* is the nominal rolling circle radius of the wheel, *x* is the arc length along the wheel surface and *h* is the effective abrasion depth. According to the geometric principle, the relationship between the depth and length of flat scars is usually converted into the relationship between the depth and the rotation angle of the wheel. Therefore, the radius of the wheel is subtracted from the depth of the flat scar to obtain the radius change of the old flat scar of the wheel, as shown in Equations (8) and (9).

(8) $$R_{\beta }=R-\frac{Ra^{2}}{8}\left[1-\cos\left(\frac{\pi }{a}\beta \right)\right]$$

(9) $$a=\frac{L}{2R}$$

According to “Railway Technical Management Regulations”, the turning repair for wheel pairs with flat scars is supposed to meet the following requirements: the depth of wheel abrasion is no more than 0.7 mm, the length and the depth of defects or peeling on the wheel tread are no more than 40 mm and 1 mm, respectively. Therefore, old flat scars with a length range of 10–20 mm were established and analyzed in this paper. Data generated using the mathematical model of flat scars were imported into the vehicle model and simulated. The excitation frequency function of flat scars is shown in Equation (10).

(10) $$f=\frac{v}{2\pi R}$$

where *v* is the vehicle speed and *R* is the wheel radius.

#### 2.2.4. Dynamic Characteristics and Signal Features of Coupled Faults

**Figure 2:**
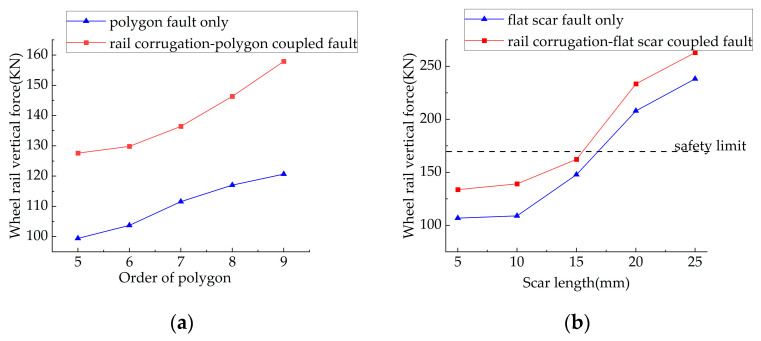
Curves of wheel–rail vertical force under different faults: (**a**) curves of wheel–rail vertical force under polygon fault and rail corrugation–polygon coupled fault; (**b**) curves of wheel–rail vertical force under flat scar fault and rail corrugation–flat scar coupled fault.

To explore the impact of wheel–rail coupled faults on the vehicle body, wheel–rail vertical force was selected as the evaluation index with a safety limit of 170 KN. The subway condition was set to variable speed at 0–50 km/h within a straight traction section. The data of wheel–rail vertical force was obtained under single faults and coupled faults including rail corrugation–polygon coupled faults and rail corrugation–flat scar coupled faults, respectively, as shown in Figure 2.

It can be clearly seen from Figure 2 that the wheel–rail vertical force under coupled faults is higher than that under single fault. A conclusion is drawn that coupled faults exacerbate the wheel–rail vertical effect, leading to further wheel–rail relationship deterioration compared to a single fault, which produces a severer impact on vehicle safety.

According to Equations (3), (5) and (9), the characteristic frequency of rail corrugation is higher while the characteristic frequencies of polygonization and flat scars are lower when the coupled fault is located in the variable-speed section. For fault signals with different and coexistent characteristic frequencies, it is difficult to identify each fault signal from coupled fault signals using traditional time–frequency analysis methods due to the complex signal characteristics.

## 3. Adaptive Parameterized Domain Mapping Method Based on Whale Optimization Algorithm

Acceleration signals of the axle box in rail vehicles under variable speed conditions possess nonstationary characteristics and are affected by strong noise, making it challenging to achieve fault feature extraction and frequency localization for diagnosis. Although order tracking is capable of overcoming the problem of frequency distortion, the instantaneous angular velocity is difficult to detect, especially in situations with strong noise or close frequency intervals. However, typical frequency distortion is caused by changes in velocity or velocity fluctuations, which means the IF of the signal component associated with the instantaneous angular velocity varies with the instantaneous angular velocity. The problem of frequency distortion is solved from another perspective by adopting the mapping between the time domain and the pseudo-time domain as a powerful tool for dealing with the frequency distortion of rotating mechanical signals.

### 3.1. Basic Theory of Parameterized Domain Mapping

The signal to be processed is mapped into a new pseudo-time domain using a PDMF [40], which eliminates the nonstationary characteristics of the signal and obtains a TFR with superior performance. The IFs of signals generated using rotating machine are proportional and the signals are modeled as MTNCMs, defined in Equation (11).

(11) $$s\left(t\right)=\sum_{i=1}^{I}s_{i}\left(t\right)$$

with

(12) $$s_{i}\left(t\right)=a_{i}\left(t\right)\cos\left[\theta_{i}\left(t\right)\right],i=1,2,\ldots ,I$$

(13) $$\theta_{i}\left(t\right)=2\pi n_{i}\int_{0}^{t}f\left(\tau \right)d\tau +\theta_{0i}$$

(14) $$a_{i}\left(t\right)>0,n_{i}>0,f\left(\tau \right)>0$$

$S_{i}\left(t\right)$ denotes the *i*th MTNCM of the signal, $a_{i}\left(t\right)$, $\theta_{i}\left(t\right)$, $\theta_{0i}$ denote the instantaneous amplitude, instantaneous phase and initial phase of the *i*th MTNCM, respectively. The IF of each MTNCM concerning the time domain is shown in Equation (15) according to definition.

(15) $$IF_{ti}=\frac{1}{2\pi }\frac{d\theta_{i}\left(t\right)}{dt}=n_{i}f\left(t\right)$$

where *n* is the relative order and *f*(*t*) is RTF. Each MTNCM has a wide band and can be processed simultaneously using domain mapping if RTF varies with time. Define

(16) ψ=r(t)

where ψ is the coordinate variable in the pseudo-time domain and *r*(·) is the monotonic domain mapping function. The IF of each MTNCM with respect to ψ is shown in Equation (17).

(17) $$IF_{\psi i}=\frac{1}{2\pi }\frac{d\theta_{i}\left(t\right)}{d\psi }=n_{i}\frac{f\left(t\right)}{r^{\prime }\left(t\right)}$$

From (17), the IFs of all MTNCMs about ψ are constants if $f\left(t\right)/r^{\prime }\left(t\right)$ is a constant. The core problem is constructing a pseudo-time domain and finding a suitable PDMF *r*(*t*) so that the signal to be processed remains smooth in the pseudo-time domain. After domain mapping, the signal in the pseudo-time domain is denoted as $S_{\psi }\left(P_{r}\right)$, where $P_{r}$ is the parameter set of the PDMF to be optimized. The signal is smooth in the pseudo-time domain if $P_{r}$ is optimal, through which the core problem is simplified into a parameter optimization problem. The WOA is adopted as the optimization method in this paper, which will be introduced in next section.

### 3.2. Optimization of Parameterized Domain Mapping

The WOA [49] was selected for the optimization of the PDM method, which has the advantages of fast convergence speed, high search accuracy, wide applicability and excellent stability.

#### 3.2.1. Whale Optimization Algorithm

The optimization process of the WOA starts with random initialization of the population and the whole search process is divided into three stages: encircling prey, bubble-net attacking and random search. The prey encirclement process can be represented by a mathematical model as shown in Equations (18) and (19).

(18) $$\vec{D}=\left|\vec{C}\times {\vec{X}}^{*}(t)-\vec{X}(t)\right|$$

(19) $$\vec{X}(t+1)={\vec{X}}^{*}(t)-\vec{A}\cdot \vec{D}$$

where *t* is the current number of iterations, ${\vec{X}}^{*}\left(t\right)$ denotes the position of the current population optimal solution, $\vec{X}\left(t\right)$ denotes the current position of the whale, $\vec{D}$ is the distance between ${\vec{X}}^{*}\left(t\right)$ and $\vec{X}\left(t\right)$, and $\vec{A}\cdot \vec{D}$ indicates an encircling step with $\vec{A}=2\vec{a}\cdot \vec{r}-\vec{a}$ and $\vec{C}=2\vec{r}$. $\vec{r}$ is a random vector uniformly distributed in the interval [0,1] and the elements of $\vec{a}$ are the control parameters that decrease linearly from 2 to 0 during the iterative process.

Bubble-net attacking consists of two strategies: shrinking encircling and spiral updating position. (a) Shrinking encircling: The reduction in the fluctuation amplitude of $\vec{A}$ is achieved by decreasing the convergence factor. When $\left|\vec{A}\right|<1$, each whale moves closer to the target prey, achieving a shrinking encirclement of the prey. (b) Spiral updating position: the same probability p is chosen for the shrinkage envelope and spiral position update and the mathematical model is represented in Equation (20).

(20) $$\vec{X}(t+1)=\left\{ \begin{array}{l} {\vec{X}}^{*}(t)-\vec{A}\cdot \vec{D}\;,p<0.5,\left|\vec{A}\right|<1\\ {\vec{D}}^{\prime }\cdot e^{bl}\cdot \cos(2\pi l)+{\vec{X}}^{*}(t)\;,p\geq 0.5 \end{array}\right.$$

where ${\vec{D}}^{\prime }=\left|{\vec{X}}^{*}\left(t\right)-\vec{X}\left(t\right)\right|$ denotes the distance between each individual in iteration *t* and the current optimal candidate solution, *b* is a constant coefficient defining the spiral form, *l* is a random number in the interval [−1,1] and *p* is a probability factor with uniform distribution in the interval [0,1]. *p* = 0.5 is adopted in this paper.

In the random search phase when *p* < 0.5 and $\left|\vec{A}\right|\geq 1$, individual whales randomly select other individual whales and move towards them. The mathematical model is represented in Equations (21) and (22).

(21) $$\vec{D}=\left|\vec{C}*{\vec{X}}_{rand}(t)-\vec{X}(t)\right|$$

(22) $$\vec{X}(t+1)={\vec{X}}_{rand}(t)-\vec{A}\cdot \vec{D}$$

**Figure 3:**
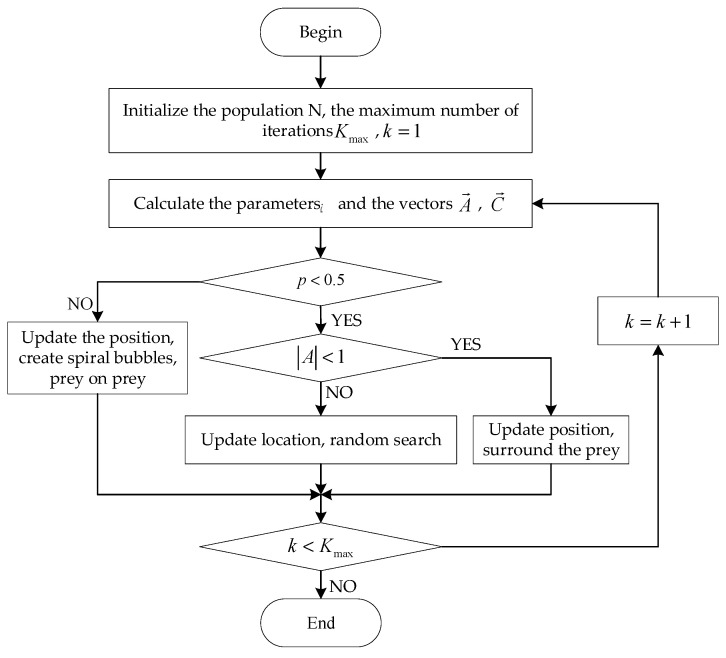
Flow chart of WOA.

The flow block diagram of the WOA is shown in Figure 3.

#### 3.2.2. Optimization Index Rényi Entropy

Rényi entropy [50] was selected to evaluate the performance of the adopted time–frequency analysis method. An important development direction of time–frequency analysis is to improve the time–frequency resolution. A TFR with high time–frequency resolution is intuitively reflected by the high energy concentration in the time–frequency plane. Therefore, high energy concentration indirectly reflects high time–frequency resolution ability to a certain extent. In time–frequency analysis, Rényi entropy can be adopted to measure the time–frequency energy concentration, which is defined in Equation (23).

(23) $$R_{\alpha }(Spec)=\frac{1}{1-\alpha }\log_{2}\iint Spec^{\alpha }(t,f)dtdf$$

where α is the order of Rényi entropy and α = 0.5 is adopted in this paper. The signal remains smooth in the pseudo-time domain and performs the highest energy concentration if the parameter set $P_{f}$ is optimal. Thus, the parameter optimization problem can be expressed in Equation (24).

(24) $${\tilde{P}}_{f}=\arg\min\underset{P_{f}}{[}R_{\alpha }(Spec(P_{f}))]]$$

The optimal value of Rényi entropy is obtained through employing the WOA to optimize the parameter set of the PDMF. Performance evaluation between different algorithms is conducted next.

### 3.3. Optimization Framework

**Figure 4:**
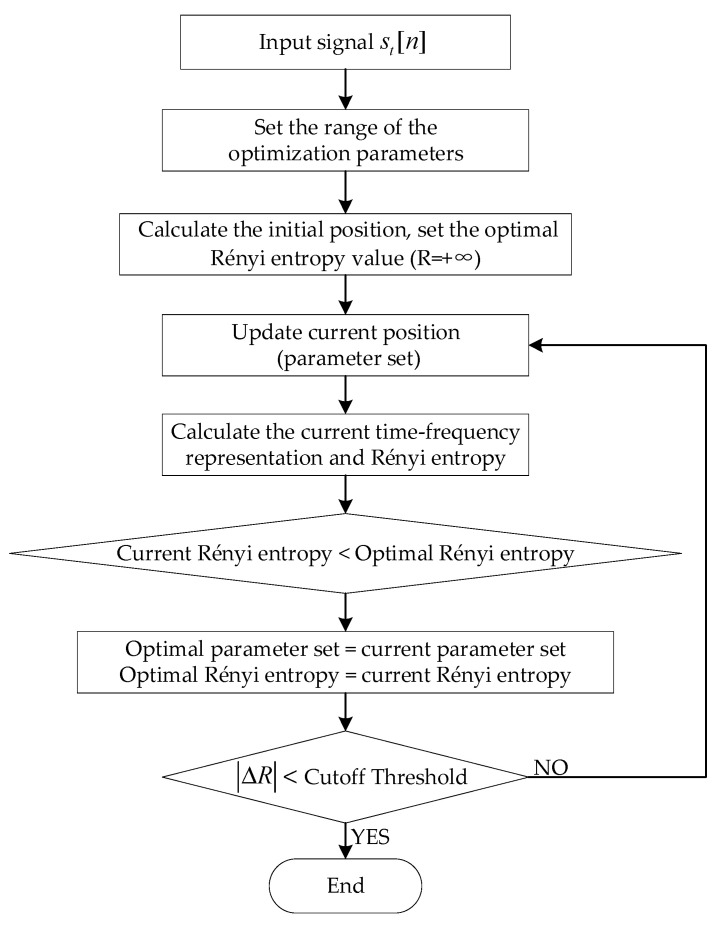
Technical flow chart of the proposed APDM method.

Acceleration signals of rail vehicles with coupled faults were analyzed based on the proposed optimization algorithm. The technical block diagram is shown in Figure 4, where the cutoff threshold is $10^{-4}$.

### 3.4. Performance Evaluation of Algorithm

PSO [51] and SSA [52] are two classic metaheuristic optimization algorithms. In this section, by comparing these two optimization algorithms with the WOA, the advantages of the WOA selected in this paper for parameter set optimization are highlighted including faster convergence speed, higher accuracy and fewer iterations.

**Figure 5:**
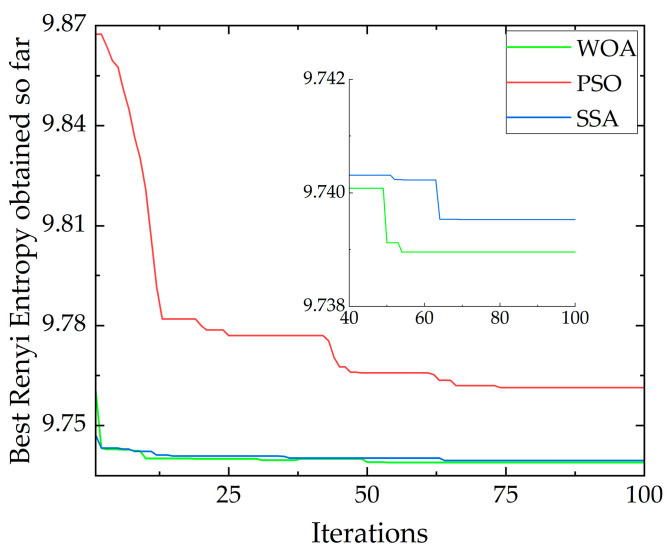
A comparison between WOA, PSO and SSA.

An axle box vibration signal within a coupled fault section with a duration of 3 s was extracted to evaluate the performance of the above three algorithms by adopting Rényi entropy as the optimization index. The population size of all three optimization algorithms was set to 30 during the process of comparison. The convergence curves of different algorithms obtained using simulation are shown in Figure 5.

As shown in Figure 5, the optimal values of Rényi entropy obtained using PSO, SSA and WOA optimization are 9.7614, 9.7395 and 9.7389, respectively. After optimization, the objective Rényi entropy obtained using the WOA was minimum and optimal. This means the convergence accuracy of the WOA is higher than that of PSO and SSA.

The numbers of iterations required for PSO, SSA and WOA to obtain the optimal objective function value, as shown in Table 1, are 73, 70 and 54, respectively. Among them, the number of iterations of the WOA is smaller than that of PSO and SSA. Compared with PSO and SSA, the number of iterations of the WOA is reduced by 26% and 23%, respectively.

## 4. Simulation and Experimental Verification

Models of two kinds of coupled faults are established in this section including rail corrugation–polygon coupled fault and rail corrugation–flat scar coupled fault, respectively. Under variable speed conditions, STFT, unoptimized PDM and APDM methods were adopted to diagnose the simulation data obtained under the above coupled faults, and their diagnostic effects were analyzed and compared.

### 4.1. Rail Corrugation–Polygon Coupled Fault Signal Analysis

#### 4.1.1. Setting of Traction Condition

**Figure 6:**
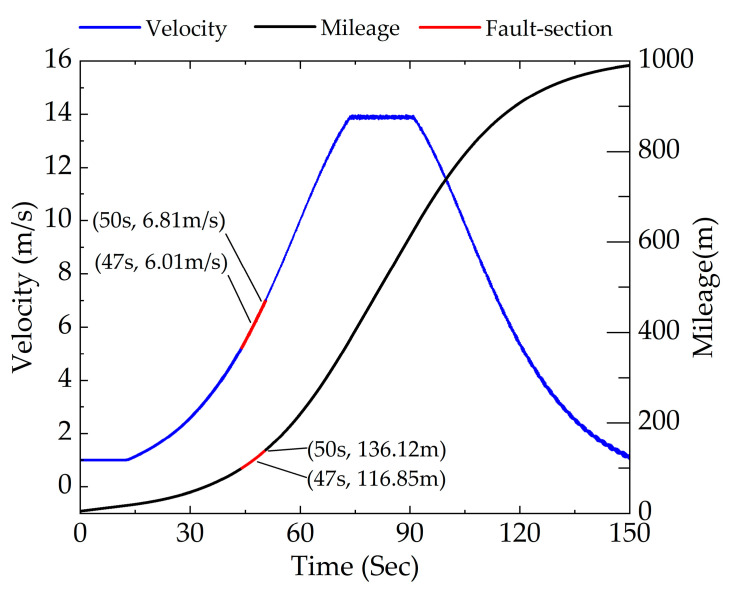
Global mileage–time and speed–time image under traction conditions.

The simulation condition was set at a straight-line condition during the subway traction stage with a full mileage length of 1000 m. The rail corrugation fault was set at a distance between 100 and 140 m that is located in the acceleration interval of 0–50 km/h as shown in Figure 6. The rail corrugation wavelength of rail vehicles in this section is set to 100 mm and the wave depth is 0.08 mm. Set the order of the wheel polygon to 8th. Intercept the signal between 47–50 s for analysis with a speed range of 6.01–6.81 m/s and a mileage range of 116.85–136.12 m located within the coupled fault section. Therefore, the time-frequency characteristics of coupled faults under traction conditions are diagnosed through analyzing the vertical acceleration signal between 47 and 50 s of the axle box.

Time frequency analysis under traction condition using different methods is performed in the next section.

#### 4.1.2. Time–Frequency Characteristics under Traction Condition

**Figure 7:**
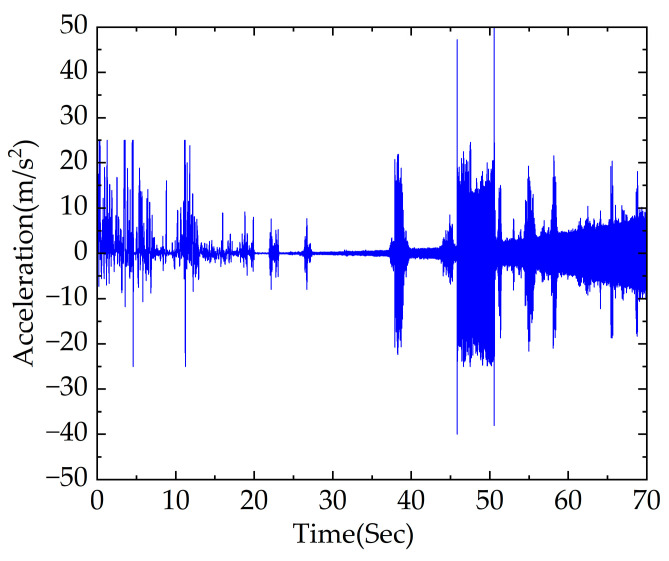
Acceleration signal of axle box in time domain under traction section.

The acceleration signal between 0 and 70 s of the axle box under the traction section using simulation was obtained, as shown in Figure 7.

It can be seen from Figure 6 and Figure 7 that the vehicle operates at a lower speed and the vertical vibration acceleration of the axle box is relatively small during the first 45 s of traction, indicating that the impact of rail corrugation excitation on the vertical vibration response of the vehicle is relatively weak, which is more affected by wheel polygon faults at this stage.

The vertical vibration response caused by rail corrugation gradually increases as the vehicle speed gradually rises. However, increased difficulty occurs in the diagnosis of rail corrugation–polygon coupled fault due to the interference of other short-wave abnormalities. It is difficult to directly distinguish the excitation of rail corrugation and wheel polygon faults from the time domain waveform and to achieve an accurate diagnosis of rail corrugation–polygon coupled fault.

The signal in time domain between 47 and 50 s within the fault section was selected for analysis. It can be concluded from Figure 6 that the range of vehicle speed in this section is 6.01–6.81 m/s. According to Equations (3) and (5), the excitation frequency of rail corrugation is 60.10–68.10 Hz and the excitation frequency of polygonization is 18.22–20.64 Hz. To be closer to the actual working condition, an acceleration signal accompanied by −10 dB Gaussian white noise is considered in this paper. STFT, PDM and APDM were adopted to analyze signals of rail corrugation–polygon coupled faults under traction conditions.

**Figure 8:**
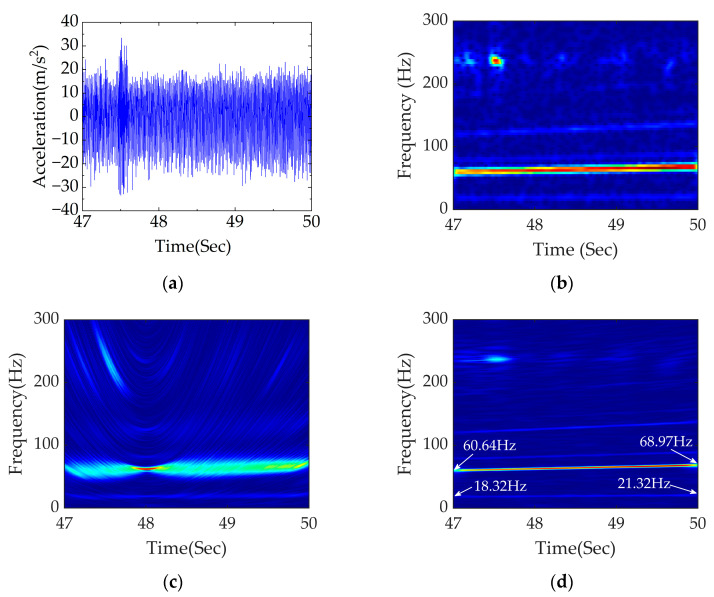
Data processing diagram group of rail corrugation–polygon coupled fault under traction conditions: (**a**) diagram of coupled fault between 47 and 50 s in time domain; (**b**) time−frequency analysis of STFT; (**c**) time–frequency analysis of PDM; (**d**) time–frequency analysis of APDM.

The simulated signal with noise is shown in Figure 8a.

Figure 8b shows the result of time–frequency analysis obtained using STFT, which is divergent in energy and sensitive to noise, making it difficult to accurately locate the core frequency. It is challenging to accurately diagnose the wavelength of rail corrugation and the order of polygon separately in coupled faults. The time–frequency analysis of PDM and APDM are shown in Figure 8c,d, respectively. It can be seen that the fault frequency location is obscure, and the performance of energy concentration is bad. In contrast, the TFR obtained using APDM delivered excellent performance including accurate fault frequency location, high energy concentration and strong noise resistance after optimization. Variable frequency signals of 60.64–68.97 Hz and 18.32–21.32 Hz can be clearly seen, which are close to the calculated theoretical range, indicating that the wavelength of rail corrugation and the order of polygon can be accurately located using APDM.

**Figure 9:**
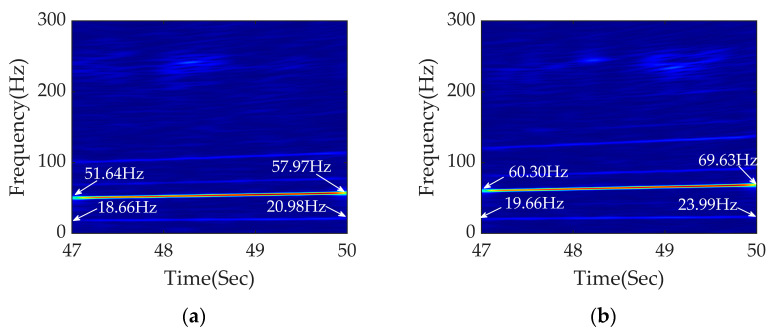
Time–frequency analysis of APDM diagnosis under traction conditions: (**a**) fault diagnosis result of rail corrugation with wavelength 120 mm and 8th polygon; (**b**) fault diagnosis result of rail corrugation with wavelength 100 mm and 9th polygon.

After the above analysis, the diagnostic effect of APDM is further validated through changing the rail corrugation wavelength and the polygon order in the next section. The results are shown in Figure 9.

Figure 9a shows the time–frequency analysis of APDM under traction conditions with a rail corrugation wavelength of 120 mm. The variable speed range is still 6.01–6.81 m/s. According to Equations (3) and (5), the excitation frequencies of rail corrugation and polygonization are 50.08–56.75 Hz and 18.22–20.64 Hz, respectively. The frequency conversion signals at 51.64–57.97 Hz and 18.66–20.98 Hz are clearly seen, which are close to the calculated theoretical range. APDM achieves excellent diagnostic effects on rail corrugation faults of different wavelengths.

Figure 9b shows the time–frequency analysis of a 9th-order polygon fault at a rail corrugation wavelength of 100 mm. The frequency conversion signals at 60.30–69.63 Hz and 19.66–23.99 Hz are clearly seen, which are close to the calculated theoretical values of 60.10–68.10 Hz and 20.49–23.22 Hz, respectively.

Thus, APDM preforms excellent diagnostic effects on diagnosing fault signals of rail corrugation with different wavelengths and polygonization with various orders under traction conditions.

#### 4.1.3. Setting of Braking Condition

**Figure 10:**
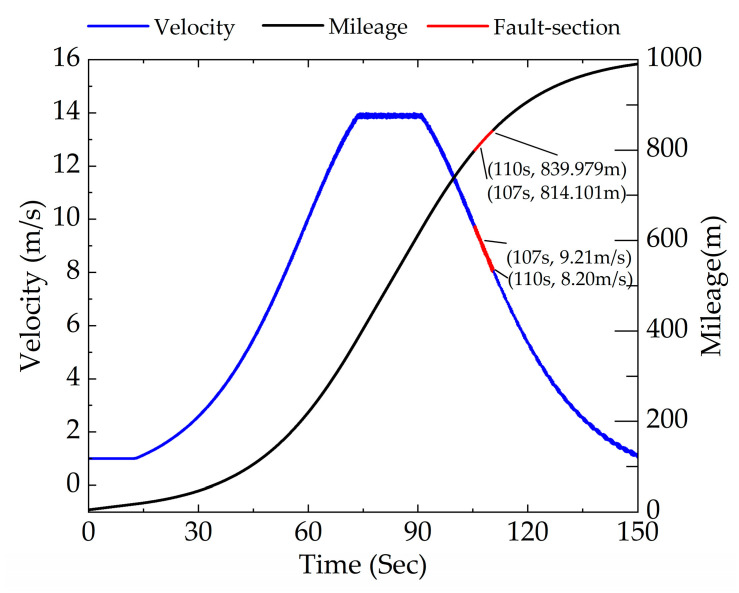
Global mileage–time and speed–time image under braking conditions.

The simulation condition was set to a straight line during the subway braking stage with a full mileage of 1000 m. The rail corrugation fault was set at a distance of 800–840 m, which is within the deceleration interval of 50–0 km/h as shown in Figure 10. The rail corrugation wavelength of rail vehicles in this section is set to 100 mm and the wave depth is 0.08 mm. Set the order of the wheel polygon to 8th. Intercept the signal between 107 and 110 s with a speed range of 9.21–8.20 m/s and a mileage range of 814.101–839.979 m located within the coupled fault section for analysis. Therefore, time–frequency characteristics of coupled faults under braking conditions can be diagnosed through analyzing the vertical acceleration signal between 107 and 110 s of the axle box.

Time–frequency analysis under braking conditions using different methods is performed in the next section.

#### 4.1.4. Time–Frequency Characteristics under Braking Conditions

**Figure 11:**
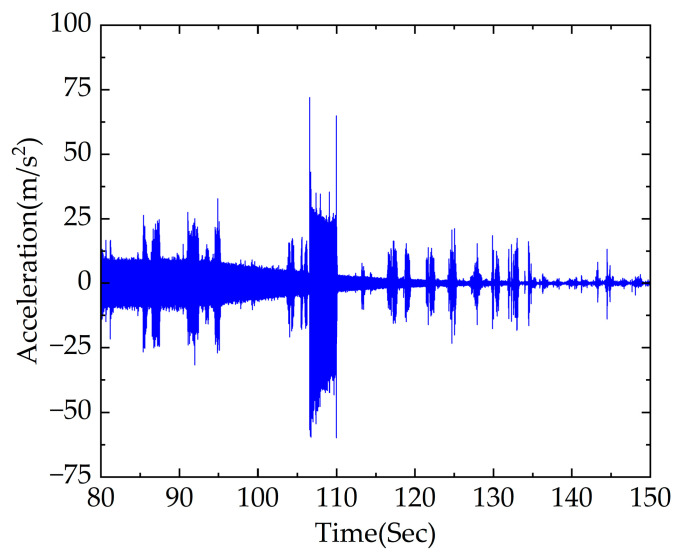
Time domain diagram of axle box acceleration under braking condition.

The acceleration signal between 80 and 150 s of the axle box under the braking section was obtained using simulation, as shown in Figure 11.

It is difficult to directly distinguish the excitation of rail corrugation and wheel polygonization separately from the time domain diagram in Figure 11, causing difficulty in achieving accurate diagnosis of rail corrugation–polygon coupled faults.

The time domain signal between 107 110 s within the fault section was selected for analysis. According to Figure 10, the range of vehicle speed is 9.21–8.20 m/s. Theoretical values of the excitation frequency of rail corrugation and polygon are 92.10–82.00 Hz and 27.92–24.85 Hz, respectively. To be closer to the actual working condition, an acceleration signal with −10 dB Gaussian white noise is considered in this section. STFT, PDM and APDM were adopted to analyze signals of rail corrugation–polygon coupled fault under braking conditions.

**Figure 12:**
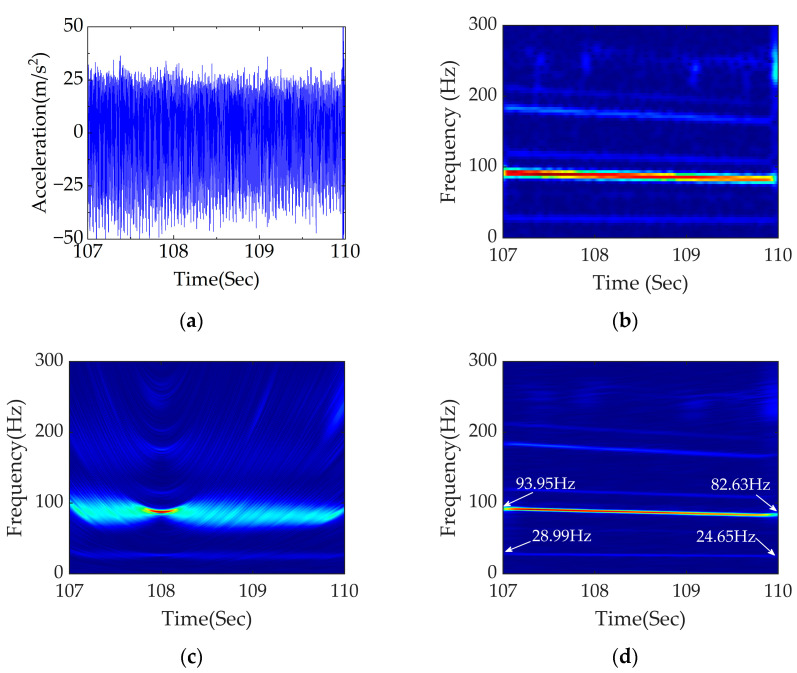
Data processing diagram group of rail corrugation–polygon coupled fault under braking conditions: (**a**) diagram of coupled fault between 107 and 110 s in the time domain; (**b**) time–frequency analysis of STFT; (**c**) time–frequency analysis of PDM; (**d**) time–frequency analysis of APDM.

The time domain diagram of the intercepted signal between 107 and 110 s, shown in Figure 12a,b, shows the time–frequency analysis obtained using STFT. The TFR obtained using STFT shows bad performance, including divergent energy and poor noise resistance, making it difficult to accurately locate the core frequency and precisely diagnose the wavelength of rail corrugation and the order of the polygon.

Figure 12c,d show the time–frequency analysis obtained using PDM and APDM, respectively. It can be seen from Figure 12c that the fault frequency location is unclear, and the energy concentration is low. However, the TFR obtained using APDM achieves excellent effect including accurate fault frequency location, high energy concentration and strong noise resistance. The frequency conversion signals at 93.95–82.63 Hz and 28.99–24.65 Hz can be clearly seen, which are close to the calculated theoretical range. Under braking conditions, the wavelength of rail corrugation and the order of polygon can be accurately located using APDM.

**Figure 13:**
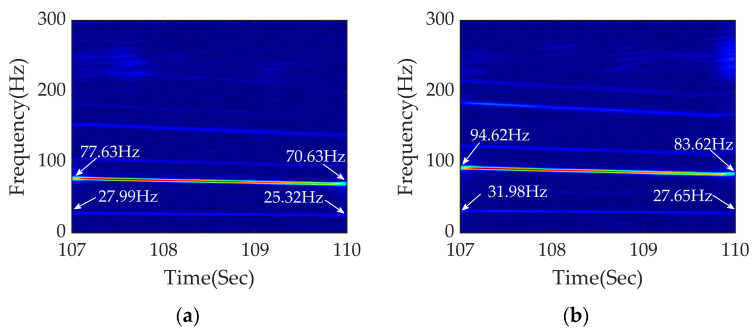
Time–frequency analysis of APDM under braking conditions: (**a**) fault diagnosis diagram of rail corrugation with wavelength 120 mm and 8th polygon; (**b**) fault diagnosis diagram of rail corrugation with wavelength 100 mm and 9th polygon.

Based on the above analysis, the effect of APDM is further validated by changing the wavelength and polygon order under braking conditions next. The results of analysis are shown in Figure 13.

Figure 13a shows the time–frequency diagram of APDM with a rail corrugation wavelength of 120 mm at a variable speed range of 9.21–8.20 m/s. Adopting Equations (3) and (5), the excitation frequencies of rail corrugation and polygonization are 76.75–68.33 Hz and 27.92–24.85 Hz, respectively. It can be seen from Figure 13a that the frequencies of fault signals are 77.63–70.63 Hz and 27.99–25.32 Hz, which are close to the calculated theoretical range. Figure 13b shows the time–frequency analysis of a coupled fault whose order of polygon is 9 and wavelength of rail corrugation is 100 mm. According to Equations (3) and (5), the excitation frequencies of rail corrugation and polygonization are 92.10–82.00 Hz and 31.41–27.96 Hz, respectively. The frequency conversion signals in Figure 13b are at 94.62–83.62 Hz and 31.98–27.65 Hz, which are close to the calculated theoretical range.

Therefore, APDM still performs well in diagnosing fault signals with different wavelengths of rail corrugation and orders of polygon under braking conditions.

### 4.2. Analysis of Rail Corrugation–Flat Scar Coupled Fault

Only fault characteristics under traction conditions are considered in this section. Set the condition at the straight-line condition within the traction stage of rail vehicles with a full mileage of 1000 m. The rail corrugation is set at 100–140 m located in the acceleration interval of 0–50 km/h. The wavelength of rail corrugation is 100 mm and the wave depth is 0.08 mm. The length of the flat scar is set to 15 mm.

The speed range in this section is consistent with that of the rail corrugation–polygon coupled fault. Therefore, a coupled fault segment of 47–50 s was selected with a speed range of 6.01–6.81 m/s. Through Equations (3) and (9), the theoretical values of the excitation frequency of rail corrugation and flat scar are 60.10–68.10 Hz and 2.27–2.58 Hz, respectively. To be closer to the actual working condition, the acceleration signal accompanied by −10 dB Gaussian white noise was considered. We analyzed the signals of rail corrugation–flat scar coupled fault under traction conditions using STFT, PDM and APDM, respectively.

**Figure 14:**
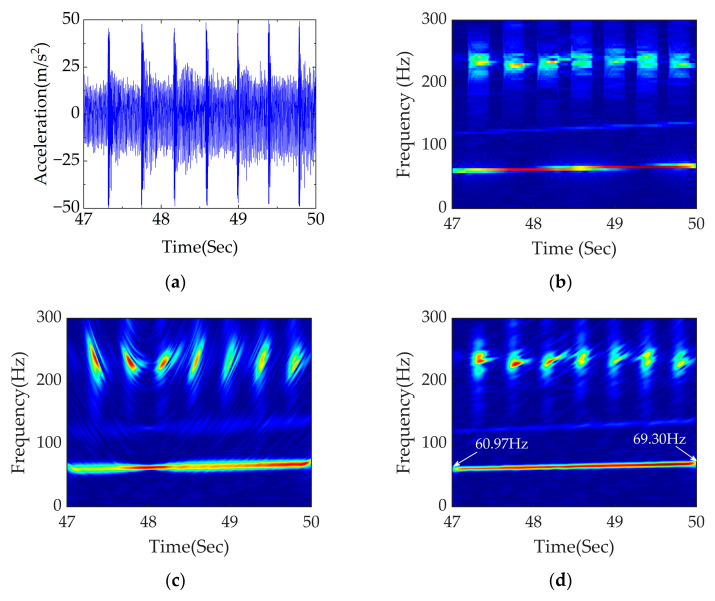
Data processing diagram group of rail corrugation–flat scar coupled fault under traction conditions: (**a**) diagram of rail corrugation–flat scar coupled fault in the time domain; (**b**) time–frequency analysis of STFT; (**c**) time–frequency analysis of PDM; (**d**) time–frequency analysis of APDM.

It is evident in Figure 14a that the impact of flat scar is huge, which has a great influence on the acceleration of an axle box. It is difficult to directly distinguish the excitation of rail corrugation from the time–domain waveform. Great difficulty occurs in diagnosing the coupled fault of rail corrugation–flat scar due to the interference of other short-wave irregularities.

Figure 14b shows the time–frequency analysis obtained using STFT. The TFR in Figure 14b represents the characteristics of divergent energy and poor interference resistance, making it difficult to accurately locate the core frequency and to precisely diagnose the wavelength of rail corrugation and the existence of flat scars. Figure 14c shows the time–frequency analysis of PDM. It can be seen that the fault frequency location is poor, and the energy concentration is low. Figure 14d shows the time–frequency analysis obtained using APDM. It can be clearly seen that the frequency conversion signal is at 60.97–69.30 Hz, which is close to the theoretical calculation range. The frequency of flat scars is relatively small, yet the high-frequency impact of a flat scar can be clearly seen. Accurate positioning of the wavelength of rail corrugation and detection of flat scars in a coupled fault are realized.

**Figure 15:**
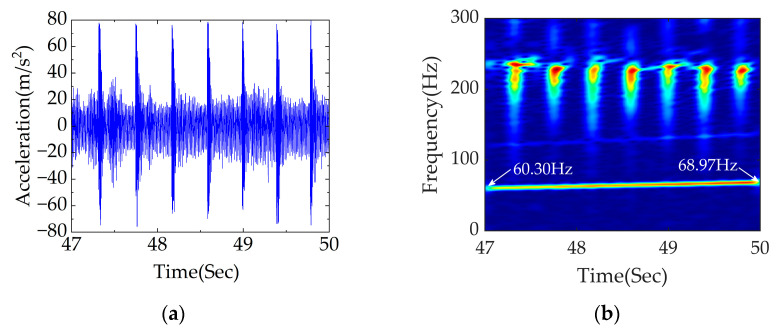
Data processing diagram group of rail corrugation–flat scar coupled fault with scar length of 20 mm: (**a**) diagram of rail corrugation–flat scar coupled fault in the time domain; (**b**) time–frequency analysis of APDM.

The diagnostic effect of APDM is further validated by changing the length of the flat scar. The results of analysis are shown in Figure 15.

Figure 15a shows the time domain diagram under traction conditions with the wavelength of rail corrugation unchanged and the length of flat scar set at 20 mm. It can be seen in Figure 15a that the flat scar has a great impact and substantial influence on the acceleration of the axle box. Figure 15b shows the time–frequency analysis obtained using APDM, which achieves accurate fault frequency localization, high energy concentration and strong noise resistance. It can be seen that the frequency conversion signal of rail corrugation is at 60.30–68.97 Hz, which is close to the theoretical range of 60.10–68.10 Hz. The frequency of flat scars is relatively small, yet the high-frequency impact of flat scars can still be obviously seen. The wavelength of rail corrugation can be accurately located and the presence of flat scars with different scar lengths can be detected using APDM.

### 4.3. Discussions and Summaries

For the diagnosis of rail corrugation–polygon and rail corrugation–flat scar coupled faults, STFT performs poorly in noise resistance and PDM performs bad in energy concentration. In contrast, the APDM method in this paper realizes stronger noise resistance and higher energy concentration for fault frequency diagnosis. APDM can diagnose the wavelength of rail corrugation, polygon order and the existence of flat scars under coupled faults with a clear core frequency of the fault, representing certain engineering application value.

### 4.4. Experimental Verification of Field-Tested Data

A field test of axle box signals was required for experimental verification. The measured acceleration signal of the axle box in a certain section of a rail vehicle’s route in a certain city was selected for the validation of APDM.

**Figure 16:**
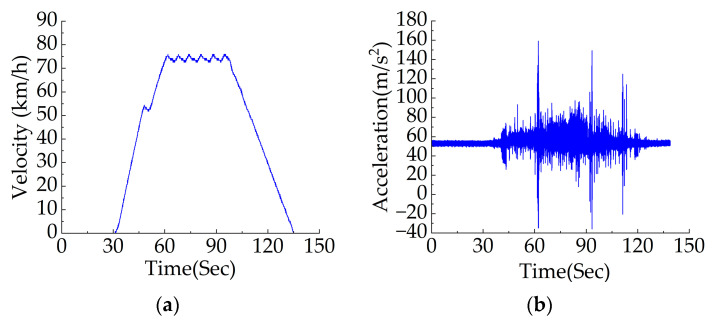
Measured speed and vibration acceleration signals: (**a**) speed–time graph of the entire section of a certain rail vehicle route; (**b**) acceleration signal of the entire section of a rail vehicle line.

Issues of frequent starting and braking exist in the actual operation of rail vehicles. Figure 16 shows that the signal testing process is in a variable speed section and numerous impact signals and noise coexist within the signal. However, it can be obviously seen that abnormal signal vibrations occur at 41–44 s and 117–121 s. The signal between 41 and 43 s within the acceleration section and the signal between 118.2 and 120.2 s within deceleration section were diagnosed using APDM.

**Figure 17:**
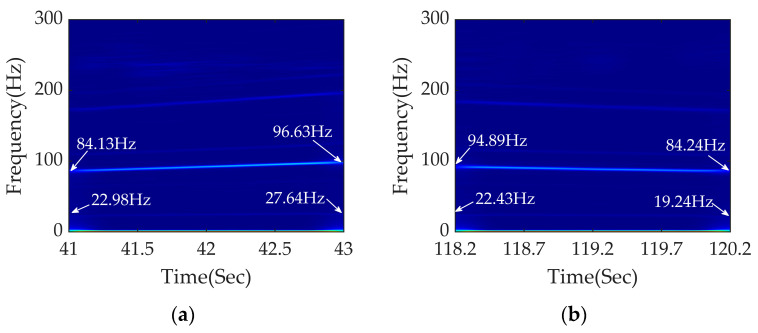
Time–frequency analysis of measured data using APDM: (**a**) time–frequency analysis between 41 and 43 s under traction conditions; (**b**) time–frequency analysis between 118.2 and 120.2 s under braking conditions.

The time–frequency analysis of the two measured sections is shown in Figure 17. It can be seen that the fault frequency diagnosed using APDM is accurate with high energy concentration and strong noise resistance. The speed ranges in Figure 16a,b are 9.00–10.56 m/s and 9.11–8.15 m/s, respectively.

According to Figure 17a, the frequency conversion signals of 84.13–96.63Hz and 22.98–27.64 Hz can be clearly seen. It can be calculated that the wavelength range of rail corrugation is 107–109 mm, which is close to the measured wavelength of 107 mm. From the signal of 22.98–27.64 Hz and the speed range, it can be concluded that the wheels have a 7th-order polygon.

According to Figure 17b, the frequency conversion signals of 94.89–84.24 Hz and 22.43–19.24 Hz can be obviously seen. It can be calculated that the wavelength range of rail corrugation is 96–97 mm, which is close to the measured wavelength of 99 mm. From the signal of 22.43–19.24 Hz and the speed range, it can be concluded that the wheels also have a 7th-order polygon.

A conclusion can be drawn that the wavelength of rail corrugation and the order of wheel polygonization can be accurately located and diagnosed using APDM under the variable speed conditions of rail vehicles. However, problems including unclear frequency ridges and insufficient accuracy still exist, which need to be enhanced in the future.

## 5. Conclusions

A dynamic model of rail vehicles under variable speed conditions was established through which the vibration signals of the axle box under coupled faults were obtained, including rail corrugation–polygon and rail corrugation–flat scars. Signals existing in actual working conditions commonly exhibit high complexity leading to difficulty in effectively setting parameters during time–frequency analysis. To overcome this problem, APDM was proposed in this paper to optimize the PDMF parameter set and improve its self-adaptability. Its advantages are as follows:

Under frequent starting and braking conditions, the proposed APDM time–frequency method can accurately analyze complex vibration signals with strong noise resistance:

(1) A comparison was carried out between PSO, SSA and WOA to evaluate their performance during the optimization process. The number of iterations of WOA adopted in this paper decreased by 26% and 23%, respectively, compared with PSO and SSA, which means that the WOA performs faster in terms of convergence speed and has a more accurate Rényi entropy value;
(2) Compared with STFT and PDM, APDM achieves the advantages of accurate fault frequency location, high energy concentration and excellent noise resistance.

APDM achieves accurate positioning of rail corrugation wavelength, determination of polygon order and accurate diagnosis of flat scars under variable speed conditions of rail vehicles. This provides a reference for coupled fault detection of rail vehicles under variable speed conditions and has certain engineering application value.

In the future, further research will be conducted on the selection of optimization indicators and optimization algorithms with faster convergence speed and higher accuracy during the optimization process of parameter sets. In addition, synchrosqueezing transform is a postprocessing method for time–frequency analysis in signal processing that stacks the energy of each time–frequency point to the energy center. Therefore, the further study of a postprocessing method for parameterized time–frequency analysis named the APDM-based synchrosqueezing transform diagnosis method can be conducted. Time–frequency analysis methods for engineering practice ensuring both excellent time–frequency clustering and reconstruction will be continued in the future.

## Figures and Tables

**Table 1:** Performance comparison of the proposed method optimized using PSO, SSA and WOA.

Algorithms	Desired Iterations	Best Rényi Entropy Obtained
PSO	73	9.7614
SSA	70	9.7395
WOA	54	9.7389

## Data Availability

Data is unavailable due to privacy or ethical restrictions.

## Nomenclature

APDM	Adaptive parameterized domain mapping.
STFT	Short-time Fourier transform.
WT	Wavelet transform.
ASTFT	Adaptive short-time Fourier transform.
TFR	Time–frequency representation.
TFA	Time–frequency analysis.
IF	Instantaneous frequency.
GPTF	Generalized parameterized time–frequency transform.
PDM	Parameterized domain mapping.
PDMF	Parameterized domain mapping function.
WOA	Whale optimization algorithm.
MTNCM	Mono-trend nonlinear chirp modes.
GD	Generalized demodulation.
TLOT	Tacholess order tracking.
PSO	Particle swarm optimization.
SSA	Sparrow algorithm optimization.
RTF	Relative trend function.
